# Gut microbiome variation in pulmonary TB patients with diabetes or HIV comorbidities

**DOI:** 10.3389/frmbi.2023.1123064

**Published:** 2023-03-15

**Authors:** Portia Abena Morgan, Prince Kofi Parbie, Desmond Opoku Ntiamoah, Augustine Asare Boadu, Prince Asare, Ivy Naa Koshie Lamptey, Cecilia Nancy Gorman, Emmanuel Afreh, Adwoa Asante-Poku, Isaac Darko Otchere, Sammy Yaw Aboagye, Dorothy Yeboah-Manu

**Affiliations:** ^1^Department of Bacteriology, Noguchi Memorial Institute for Medical Research, University of Ghana, Accra, Ghana; ^2^West African Center for Cell Biology of Infectious Pathogens, University of Ghana, Accra, Ghana; ^3^AIDS Research Center, National Institute of Infectious Diseases, Tokyo, Japan; ^4^Rush University Medical Center, Department of Microbial Pathogens and Immunity, Chicago, IL, United States; ^5^Institute for Environment and Sanitation Studies, University of Ghana, Accra, Ghana

**Keywords:** tuberculosis, diabetes, HIV/AIDS (acquired immunodeficiency syndrome), comorbidity, coinfection, microbiome

## Abstract

**Background:**

The gut microbiota is known to play a critical role in shaping the host immunity, and metabolism and influences the onset and progression of both communicable and non-communicable diseases. This study assessed the gut microbiome of tuberculosis (TB) cases with diabetes mellitus (DM) or HIV comorbidities before anti-TB therapy and after the intensive phase anti-TB therapy.

**Methods:**

Ninety cases comprising 60 TB-only, 23 TB-DM, 7 TB-HIV were recruited, among which 35 TB-only, 10 TB-DM, 5 TB-HIV were also sampled after 2 months of anti-TB treatment. Total gut microbiome was detected by 16S rRNA gene sequencing of DNA extracted from collected stool specimen. The taxonomic and functional diversity of the different groups were compared in addition to changes that could occur after 2 months antibiotics use.

**Results:**

Compared to the healthy controls, the gut microbiome of all the TB cohorts was characterized by a significant decreased alpha diversity and significant compositional changes. All the three TB cohorts were enriched with inflammatory related microorganisms of the genera *Escherichia-shigella*, *Streptococcus*, *Enterococcus* and *Erysipelatoclostridium* with depletion in beneficial taxa of the genera *Faecalibacterium*, *Bifidobacterium* and *Clostridium*. In pairwise comparison with the healthy controls, the TB-only cohort were enriched with *Streptococcus* and *Erysipelatoclostridium*, the TB-DM enriched with *Bacteroides*, and TB-HIV enriched with *Escherichia-shigella, Dialister* and *Erysipelatoclostridium*. After the intensive phase anti-TB therapy, there was general enrichment of the genera *Erysipelotrichaceae_*UCG 003*, Veillonella* and *Fusobacterium*.

**Conclusion:**

Our findings show a dysbiotic gut microbiome and associated upregulation of inflammation related microorganism in gut microbiome of TB individuals with or without comorbidity.

## Introduction

Tuberculosis (TB), caused by the *Mycobacterium tuberculosis* complex (MTBC) is an ancient disease and the 13^th^ cause of death globally (WHO, 2021). Only about 5 -10% of infected individuals progress to active TB disease in their lifetime; identified risk factors that may hasten progression to diseases (Flynn and Chan, 2001) include malnutrition, excessive alcohol consumption, smoking, HIV- coinfection, and diabetes mellitus (DM) (Narasimhan et al., 2013). According to the 2021 global TB report, among the 10 million new infections recorded, 8.2% were coinfected with HIV whereas 16% were comorbid with DM (WHO, 2021).

The gut microbiome, described as the last organ of the human body due to specific biochemical interaction and systemic integration with the host, comprises all microbial community and their genes inhabitant in the gut (Dumas et al., 2018; Kim and Benayoun, 2020). Gut microbes perform immunomodulatory role by sending out signals that promote immune cell maturation, influencing infection risks and disease progression (Maji et al., 2018; Hu et al., 2019). Short chain fatty acids (SCFA) produced by some of the normal flora of the gut (Makki et al., 2018) supports maintenance of intestinal barrier integrity and guard the host against infection and colorectal cancer (Peng et al., 2009; Lewis et al., 2010; Besten et al., 2013). The interaction between the gut microbes and the immune system is extremely homeostatic and carefully regulated such that in a disturbed and dysbiotic state the host immunity is negatively impacted (Honda and Littman, 2016).

The gut microbiota also influences distal organs such as the lungs through the gut-lung axis; early life gut depletion in *Faecalibacterium*, *Lachnospira*, and *Rothia* has been linked to asthma in humans (Barcik et al., 2020). Also, the gut microbiota has been shown to boost respiratory defenses through signaling granulocyte-macrophage colony-stimulating factor (GM-CSF), which accelerates pathogen clearance by alveolar macrophages *via* extracellular signal-regulated kinase signaling (Brown et al., 2017). Gut dysbiosis due to infections and/or use of antimicrobials damages this gut related host response predisposing the host to illness (Sood et al., 2018). Drug susceptible TB treatment includes narrow acting Isoniazid (H), Pyrazinamide (Z), Ethambutol (E) and broad-spectrum Rifampicin (R), (collectively termed (HZRE)), acts against a wide range of gram positive and negative organisms (Diallo et al., 2021). TB treatment is considered one of the longest antibiotic regimen globally taking a minimum of six months comprising an intensive 2-months (HRZE) phase and a continuous 4-months phase of H and R (CDC, 2016). The interplay between TB and other comorbidities such as DM and HIV infection and their impact of the gut microbiome remains unclear, also the specific effects of multiple antimicrobials in both comorbid and non-comorbid.

In this study, we analyzed the variation in gut microbiome of TB patients with and without comorbidities of either HIV or DM before the start of anti-TB therapy and after the month-2 intensive phase of TB therapy. The taxonomic and functional diversity of gut microbiome of the analyzed groups were compared in addition to changes that could occur after 2 months antibiotic use in TB patients.

## Methods

### Ethics

Ethical clearance for the study and its protocols was obtained from the Institutional Review Board (IRB) of the Noguchi Memorial Institute for Medical Research (NMIMR), University of Ghana (Federal wide assurance number: FWA00001824) and the Ethics Review Committee of the Korle-Bu Teaching Hospital. Informed written consent was obtained from all participants and their respective families.

### Study participants and sample collection

Gene Xpert MTB-RIF (Cepheid, USA) and chest X-ray confirmed pulmonary TB patients from a tertiary facility and a Regional Hospital in Ghana were recruited for the study. Demographic and clinical data including age, sex, history of TB, TB bacterial load, alcohol and cigarette use, previous medication and other underlining conditions were recorded using a structured questionnaire. Healthy close household contacts for healthy controls (HC) included non-active TB, non-HIV infected, and non-diabetic adults from the same household of the cases. TB confirmed participants were screened at baseline for diabetes (fasting blood sugar above 7mmol/L and HbA1c confirmatory test above 6%) and HIV ((positive rapid First Response HIV1-2.0 card test, Premier Medical Cooperation Limited, India) & positive confirmatory OraQuick rapid test (OraSure Technologies, USA)) after which stool samples were collected following standard procedures (Thomas et al., 2015). A second sample was collected 2 months after the intensive phase of anti-TB therapy (follow-up) from the case participants, whereas a one-time point sample was collected from the HC. At month 2 follow-up, sputum conversion was examined by microscopy. Only pulmonary TB patients above 18 years were enrolled in this study. Extrapulmonary TB cases and treatment defaulters were excluded from the study.

### Amplification and 16SrRNA gene sequencing

Fresh stool samples (2.0 ml/aliquot) were frozen and stored at -80°C prior to extraction. Microbial DNA from fecal samples was extracted using QIAGEN DNeasy PowerLyzer Power Soil kit (QIAGEN, USA) per manufacturer’s instructions. The captured DNA was then washed and eluted from the membrane and stored at 4°C for downstream analysis.

The V3 and V4 region of the bacterial 16S ribosomal RNA (rRNA) gene was amplified by PCR using the primers (16S rRNA_V3. V4-F 5’- ACACGACGCTCTTCCGATCT CCTACGGNGGWG -3’ and V3. V4-R 5’- GACGTGTGCTCTTCCGATCT GACTACHVGGTATCTAATCC -3’). PCR reactions were performed using Roche® (Roche Sequencing Solutions, USA). Amplicons with the desired 450bp size after gel electrophoresis were purified and sequenced by the Illumina Miseq (Illumina, n.d). Using MiSeq reagents V3 & V4, 16S library preparation was performed based on Nextera XT DNA library Preparation kits (Illumina Inc, Hayward, CA, USA) (Illumina, n.d). Amplification of variable region 3 & 4 (V3&V4) of the 16S rRNA, barcoding, and pulling of amplicons were performed per manufacturer’s instructions (Muinck et al., 2017).

### Data analysis

The Quantitative Insights into Microbial Ecology 2 (QIIME 2 version 2021.8) (Bolyen et al., 2019) was used to quality filter, denoise and analyze the sequences (available at the NCBI Sequence Read Archive (SRA) with study ID PRJNA876282). Demultiplexed paired-end sequence reads were denoised with DADA2 into amplicon sequence variants (ASV). Samples with < 10,000 total reads were excluded from downstream analyses. Feature table and feature data summaries were generated to determine the sequence distribution per sample as well as α-diversity (abundance of ASV within a sample) and β-diversity (microbial composition between samples) analysis of the samples. Taxonomic classifications were performed using Naïve Bayesian classifier (Ziemski et al., 2021) against the SILVA database release 138 (McLaren, 2020) trimmed to the V3-V4 region of the 16S rRNA gene.

Differentially abundant taxa were identified using the linear discriminant analysis (LDA) effect size (LEfSe) (Segata et al., 2011; Mandal et al., 2015). To study the predictive functions of the gut microbiome, Phylogenetic Investigation of Communities by Reconstruction of Unobserved States (PICRUSt) (Langille et al., 2013) was used to determine the predictive microbial metagenome functional abundance from 16S rRNA sequences. Subsequently predicted functional abundance were analyzed with ANOVA-like Differential Expression tool for high throughput sequencing data version 2 (ALDEx2) (Fernandes et al., 2013) to determine differentially abundant functional pathways. For all differential abundance analyses, rare features were removed by, as previously described (ANCOM II) (Lin and Peddada, 2020).

Statistical analysis was performed using R 3.6.0 packages and GraphPad Prism version 9.1.1. For categorical variable comparison between groups, Fisher exact test was used and Wilcoxon rank-sum test or Kruskal Wallis test used for continuous variable. Multiple test correction was performed with Benjamini, Krieger and Yekutieli FDR correction. Statistical significance was determined at a *p* value less than 0.05.

## Results

### Clinical characteristics of participants

**Figure 1:**
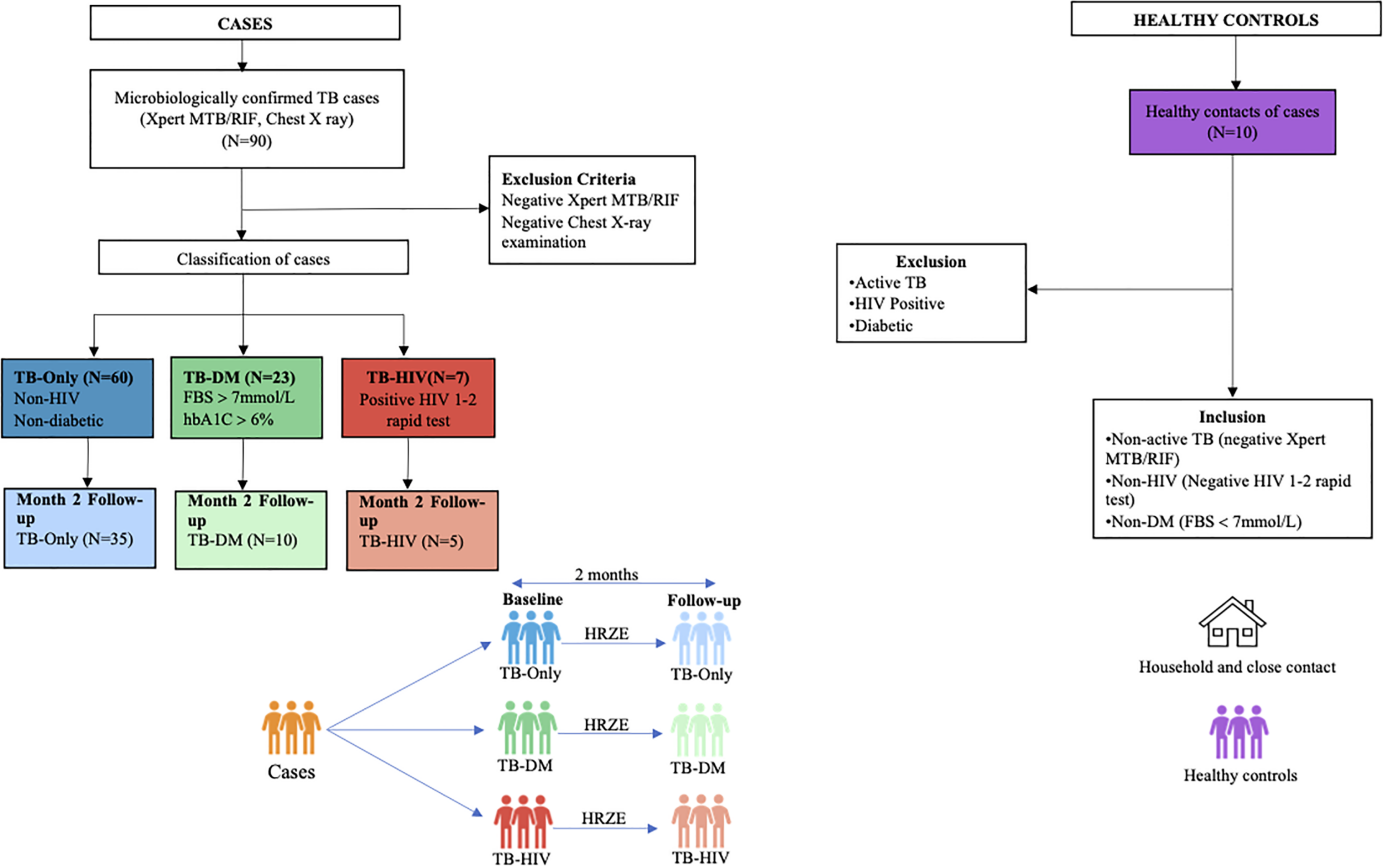
Profile of study population. Stool samples were collected from confirmed TB cases from different cohorts at baseline (60 TB-Only (without HIV/diabetes comorbidities), 23 TB-DM comorbidity, 7 TB-HIV comorbidity). After month 2 HRZE therapy cases included (52 TB-Only, 8 TB-DM, 5 TB-HIV) and 10 Healthy control.

Ninety participants made up of 60 TB-only, 23 TB-DM, 7 TB-HIV and 10 healthy controls were recruited into the study, while month-2 samples were collected from only 50 (35 TB-only, 10 TB-DM and 5 TB-HIV) of the primary cases (**Figure 1**). Seven of the cases died by 2 months after diagnosis with remaining lost to follow-up. The median age of all recruited participants was 42 years (IQR, 28.5-54.5) which was same as that for males, while the median age for females was 43.5 (**Table 1**). Out of the total baseline cases 83.3% experienced weight loss, 70% had night sweats and chest pain, and 65.5% had extreme cough (**Supplementary Table 1**).

**Table 1:** Biographical and Clinical data of participants.

Parameter	Number of Patients	Male	Female
Median age		42	43.5
Diabetics HIV positive	23 7	18 (78.3%) 5 (71.4%)	5 (21.7%) 2 (28.6%)
Total Primary cases TB – Only TB – Diabetes TB – HIV	90 60 23 7	70 (77.8%) 47 (78.3%) 18 (78.3%) 5 (71.4%)	20 (22.2%) 13 (21.7%) 5 (21.7%) 2 (28.6%)
Follow-up cases TB – Only TB – Diabetes TB – HIV Deceased	50 35 10 5 7	38 (76%) 27 (77.1%) 8 (80%) 3 (60%) 4 (57%)	12 (24%) 8 (22.9%) 2 (20%) 2 (40%) 3 (43%)
Bacterial load (GeneXpert) High Medium Low	55 17 18	40 (72.7%) 13 (76.5%) 17 (94.4%)	15 (27.3%) 4 (23.5%) 1 (5.6%)
Healthy controls Median age FBS (below 7mmol/L)	10	3 (30%) 38 3 (100%)	7 (70%) 42 7 (100%)

### Overview of the gut microbiome composition of study participant

**Figure 2:**
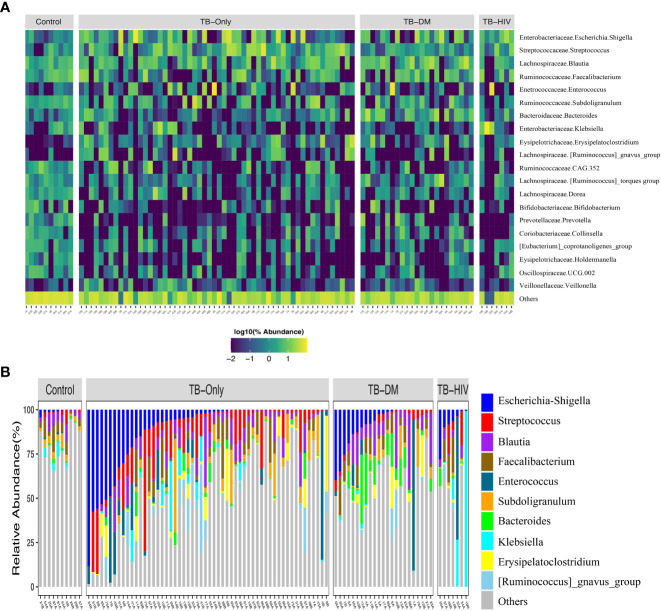
Overview of abundant gut microbial taxa in the study. **(A)** Heatmap showing top 20 predominant genera in healthy controls compared to TB patients. Each bar represents the frequencies of abundant genera for each participant in the different groups. Taxa labels are formatted as “Family.Genus”. **(B)** Taxa bar plot showing top 10 abundant genera in all three TB groups to healthy controls. TB-Only (n=56), TB-DM (n=23), TB-HIV individuals (n=7) and healthy controls (n=10).

In both cases and healthy controls, *Firmicutes* was the most abundant phylum. Among the healthy controls, *Firmicutes* (76.4%) was driven mostly by the class *Clostridia*, and families *Lachinospiraceae* (21%) and *Ruminococcaceae* (16%) (**Supplementary Figure 1**, **Supplementary Table 2**). The most abundant genera in the controls were *Faecalibacterium* (6%), and *Subdoligranulum* (3.6%) of the *Ruminococcaceae* family, *Blautia* (4.8%), and *Agathobacter* (4%) of the family *Lachinospiraceae*, *Bifidobacterium* (4.2%) of the *Actinobacteriota* phylum and *Bifidobacteriaceae* family (**Figure 2A** and **Supplementary Table 2**).

Among all the three TB cohorts, abundance in the *Firmicutes* was driven by genera *Streptococcus* (>5%), and *Enterococcus* (>5.3%) of the class *Bacilli* whilst abundance in the *Proteobacteria* phylum was driven by *Escherichia-Shigella* (>8.7%) and *Klebsiella* (> 19%) genera of the class *Gammaproteobacteria* and family *Enterobacteriaceae* (**Figure 2B** and **Supplementary Table 2**). Comparison of the 20 most abundant genera revealed great disparity among the TB cohorts and the healthy controls with the genera *Clostridia* UCG-014, *Romboutsia*, *Oscillospiraceae-*UCG-002, *Ruminococcaceae*-CAG-352, *Dorea*, *Collinsella*, *Holdermanella*, and *Lachnospiraceae torques* depleted in all the TB cohorts compared to the healthy controls (**Figure 2A**).

### Diversity of gut microbiome among the TB cohorts and healthy controls

**Figure 3:**
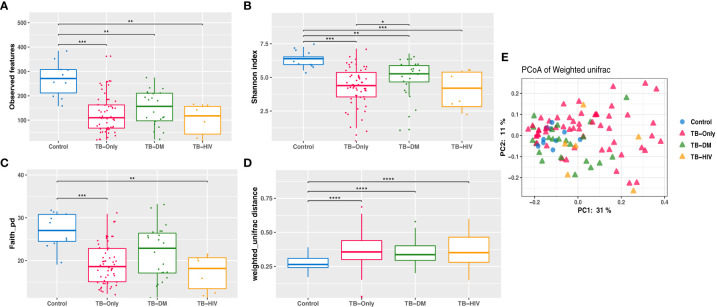
Reduced Gut microbiome diversity among TB patients at baseline. **(A)** Observed features **(B)** Shannon diversity **(C)** Faith phylogenetic diversity between all TB cohorts. * = (0.05 - 0.01), ** = (0.001 – 0.001), *** = (0.001 – 0.0001), **** = (< 0.0001). **(D)** Comparing weighted unifrac distances within groups **(E)** PCoA of weighted Unifrac between all TB cohorts where larger distance between groups indicate great variation between the groups.

Analysis of richness using observed features to determine the number of features present within the microbial community showed significantly lower richness (P < 0.01) in all TB cohorts (TB-only, TB-DM, TB-HIV) at baseline than the healthy controls (**Figure 3A**). The Shannon index and Faith phylogenetic diversity showed significant reduction (P < 0.01) in alpha diversity in all three baseline study TB cohorts compared to the healthy controls (**Figures 3B, C**).

Assessment of β-diversity by weighted Unifrac revealed significant differences (P < 0.0001) between TB groups and controls (**Figure 3D**). Further corroborated by gut microbiome compositional segregation as shown in Principal coordinates of analysis (PCoA) of weighted Unifrac distances (**Figure 3E**). Among the healthy controls there was no significant difference in diversity and abundance between the males and females using both alpha and beta diversities.

**Figure 4:**
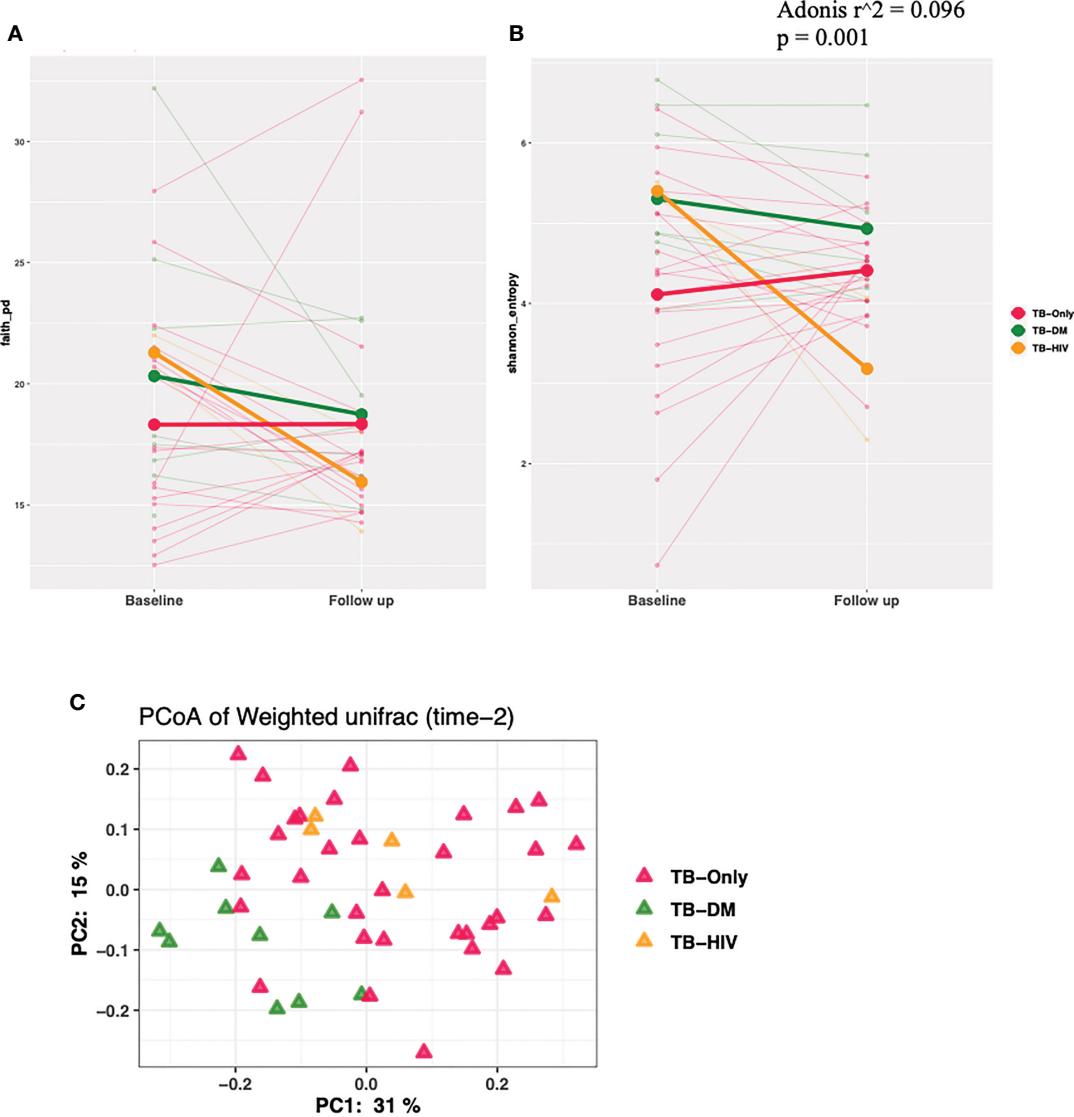
Changes in diversity in TB cohorts after 2 months HRZE. **(A)** α-diversity based on Faith phylogenetic diversity and **(B)** Shannon entropy for all TB cohorts at 2-month follow-up after HRZE compared to baseline **(C)** PCoA of weighted Unifrac at month 2.

We observed a trend of decreasing alpha diversity after 2 months of HRZE intervention in TB-DM and TB-HIV patient groups compared to baseline respectively, though no significance was detected (**Figures 4A, B**). Also, Beta diversity analysis showed no significant observable distinction in microbial composition between the groups (**Figure 4C**).

### Gut microbiome composition of specific TB cohorts

**Figure 5:**
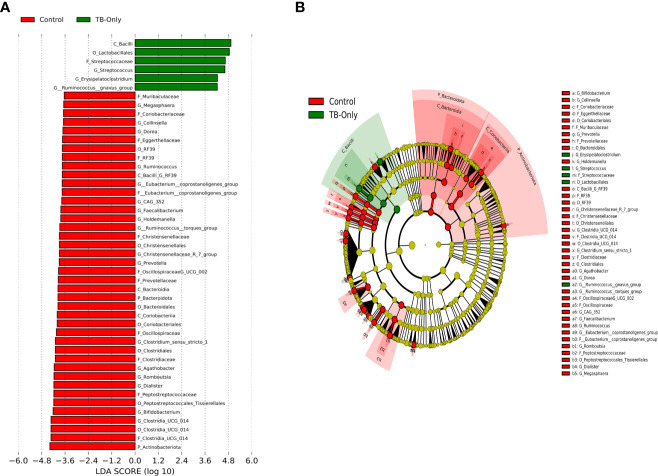
Difference in abundant taxa at baseline between TB-only and healthy controls determined by Linear discriminate analysis (LDA) effect size (LEfSe) **(A)** Discriminative features detected at 3.6 threshold and alpha of 0.01. P_Phylum, C_Class, O_Order, F_Family, G_Genus **(B)** Cladogram of all TB-only and healthy controls. Features in red and green are differentially abundant in controls and TB-Only patients respectively.

**Figure 6:**
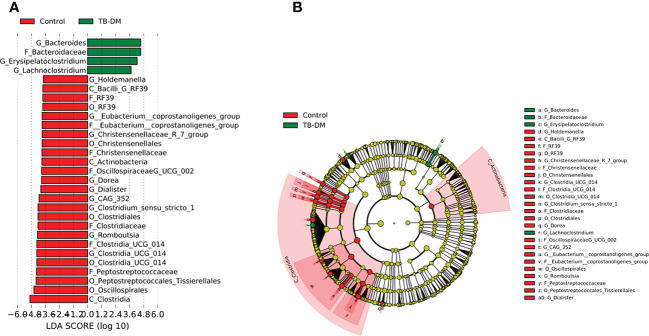
Difference in abundant taxa at baseline between TB-DM and healthy controls determined by Linear discriminate analysis (LDA) effect size (LEfSe). **(A)** Discriminative features detected at 3.6 threshold and alpha of 0.01. P_Phylum, C_Class, O_Order, F_Family, G_Genus **(B)** Cladogram of all TB-DM and healthy controls. Features in red and green are differentially abundant in controls and TB-DM patients respectively.

**Figure 7:**
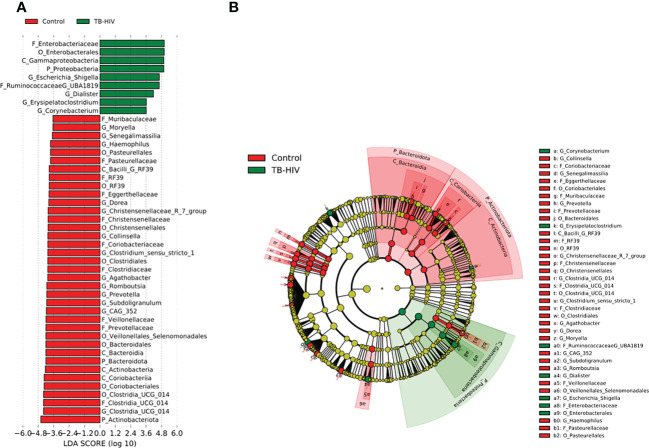
Difference in abundant taxa at baseline between TB-HIV and healthy controls determined by Linear discriminate analysis (LDA) effect size (LEfSe). **(A)** Discriminative features detected at 3.6 threshold and alpha of 0.01. P_Phylum, C_Class, O_Order, F_Family, G_Genus **(B)** Cladogram of all TB-DM and healthy controls. Features in red and green are differentially abundant in controls and TB-HIV patients respectively.

In the TB-only cohort, the class *Bacilli* (26.92%) of the phylum *Firmicutes* was significantly abundant, and this was driven by the order *Lactobacillales* (17.4%), family *Stretococcaceae* and genus *Streptococcus* (9.5%) (**Figure 5A**). In addition, the genera *Erysipelatoclostridium* and *Gamella* of class *Bacilli* was also abundant (**Figures 5A, B** and **Supplementary Figure 2**). Among the TB-DM cohort compared to the healthy controls, the family *Bacteriodaceae* and genus *Bacteriodes* (7.5%) was significantly abundant in addition to the genera *Erysipelatoclostridium* and *Lachnoclostridium* of the firmicutes phylum (**Figures 6A, B**). In the TB-HIV cohort, there was significant abundance of the phylum *Proteobacteria* (28.9%) driven by the class *Gammaproteobacteria* (28.1%), family *Enterobacteriaceae* and genus *Escherichia-Shigella* (8.7%), and *Klebsiella* (19.1%) (**Figure 7** and **Supplementary Table 2**). Also abundant was the *Butyriccoccus*, *Clostridium sensu stricto* and *Romboutsia* genera (**Supplementary Figure 2**).

### Difference in microbial composition after intensive phase anti-TB therapy

**Figure 8:**
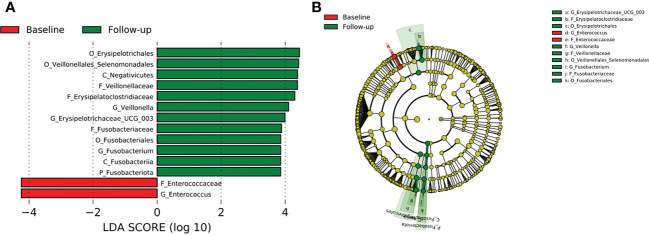
Difference in abundant taxa at baseline and month 2 HRZE treatment follow-up for all TB groups, determined by Linear discriminate analysis (LDA) effect size (LEfSe). **(A)** Discriminative features detected at 3.6 threshold and alpha of 0.01. P_Phylum, C_Class, O_Order, F_Family, G_Genus **(B)** Cladogram of baseline and follow-up. Features in red and green are differentially abundant in baseline and follow-up samples respectively.

Comparison of all the three TB cohorts before the start of TB therapy and after the 2-months intensive phase anti-TB therapy identified abundance of the phylum *Fusobacteriota* driven by the genera *Fusobacterium* of the family *Fusobacteriaceae* (**Figure 8**). In addition, the genera *Veillonella* and *Erysipelotrichaceae*_UCG 003 were significantly enriched after HRZE intervention (**Figure 8**). However, within group follow-up analysis did not reveal any significant changes in gut microbial taxa.

### Gut microbial functional prediction in TB cohorts and healthy controls

**Figure 9:**
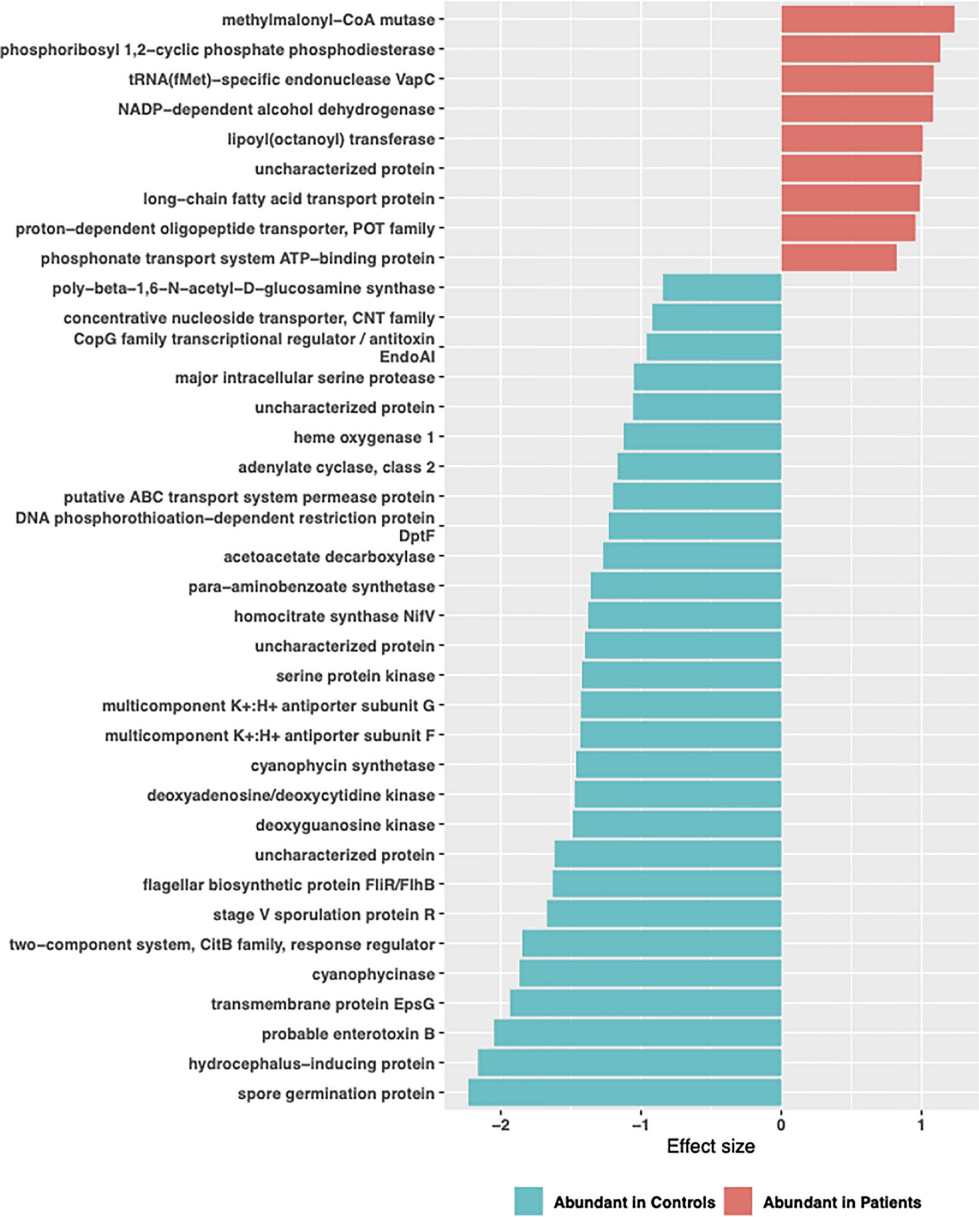
Significant differentially abundant predictive KEGG ortholog functional pathways. PICRUSt2 was used to determine the predictive microbial metagenome functional abundances from 16S rRNA sequences. Predicted functional abundances were analyzed with ALDEx2 to determine differentially abundant functional pathways.

Functional profiling was performed on baseline 16S rRNA sequences using PICRUSt2. A total of 4479 genes with 1350 enzyme classification numbers were predicted and mapped into 252 metabolic pathways. Discriminating functional features were detected by ALDEx2. No functional feature was detected to be significantly enriched or depleted when all TB patients were compared to controls (**Supplementary Figure 4**). However, when stratified by comorbidity groups, 37 KEGG ortholog features and 1 MetaCyc features were detected to be differentially abundant in TB-DM patients compared to controls (**Supplementary Figure 3**). Enriched KEGG features included features mapped methylmalonyl-coA-mutase, phosphoribosyl,1-2 cyclic phosphate phosphodiesterase, tRNA(fMet)-specific endonuclease Vap C, NADP-dependent-alcohol dehydrogenase (**Figure 9**). Metabolic function involved in isopropanol biosynthesis was detected to be significantly depleted in TB-DM patients.

## Discussion

The human gut microbiome is critical for the development of the host immune system and tissue homeostasis (Chunxi et al., 2020). Aside from the numerous beneficial roles of the microbiome within an individual, its role in the development of metabolic and infectious diseases in an altered (dysbiotic) state has been documented (Guinane and Cotter, 2013). In this study we explored the gut microbiome dynamics in TB patients with or without HIV or diabetes comorbidities before and after intensive phase anti-TB therapy. We found that the microbiome of the TB patients was enriched with inflammatory related microbial organisms with a general increase in these organisms after at 2 months HRZE intervention.

Several studies have reported a decreased gut microbiome diversity in TB patients (Luo et al., 2017; Hu et al., 2019; Cao et al., 2021; Wang et al., 2022), similar to findings in our study which shows a significantly lower gut microbial diversity in TB patients before HRZE intervention compared to healthy controls (**Figures 3A–C**). This reduced alpha diversity persisted after 2 months of HRZE intervention especially in the comorbid cases (**Figures 4A, B**). This could be indicative that in addition to TB, compositional shifts could be occurring in a TB-comorbidity specific manner.

Firmicutes are known to be a predominant phylum in the human gut (Qin et al., 2010; Arumugam et al., 2011; Morton et al., 2015). Likewise in this study, the most abundant phylum in both healthy controls (76.3%) and all TB patients (> 65.8%) was the phylum *Firmicutes* (**Supplementary Figure 1**). Several bacteria groups belonging to *Firmicutes* could utilize carbohydrates through anerobic fiber fermentation to produce short-chain fatty acids (SCFAs), such as acetates, propionate, and butyrate. These exert immunomodulatory effects including anti-inflammatory and regulation of gut’s pH to improve the availability of calcium (TeitelbaumAllan Walker, 2002; Broekaert et al., 2011; Venegas et al., 2019). Consistent with their beneficial role in the human gut, we detected an abundance of *Firmicutes* driven mostly by the genera *Clostridium, Faecalibacterium, Blautia* and *Agathobacter* in the gut of the healthy individuals (**Figure 2A** and **Supplementary Table 2**). Additionally, other genera such as *Romboutsia*, CAG-352, *Subdoligranulum*, and *Dialister* (**Figure 2A**) were also among the top 20 abundant genera in the healthy individuals. Several bacteria groups belonging to the *Ruminococcaceae* and *Lachnospiraceae* families (**Figure 2A**) which includes major human butyrate producing gut bacterial species (Venegas et al., 2019) were conspicuously low in TB patients. However, there was an abundance in other *Firmicutes* of the genera *Streptococcus*, *Enterococcus*, *Bacteriodes* and *Erysipelatoclostridium* in all the TB patients (**Figures 1A, B**).

In the TB-DM cohort, the genus *Bacteroides* was significantly abundant (**Figure 6**), comparable to studies which reported a positive association of *Bacteroides* with diabetic individuals (Sircana et al., 2018; Sharma and Tripathi, 2019; Gurung et al., 2020). The *Bacteroides* genera are beneficial for glucose metabolism as well as regulating the expression of tight junction genes in the colon to reduce gut permeability (Yoshida et al., 2018; Gurung et al., 2020). In the TB-Only cohort, the *Streptococcus* and *Erysipelatoclostridium* (**Figure 5**) which have been associated with high inflammatory conditions (Zeng et al., 2017; Wang et al., 2020), was abundant while in the TB-HIV in addition to *Erysipelatoclostridium*, *Escherchia-Shigella*, *Dialister* and *Corynebacterium* were abundant (**Figure 7**). Even though most of these bacteria are beneficial in maintaining a healthy gut microbiome, their association with inflammatory conditions could pose some health risk when it is overly abundant in the gut (Wang et al., 2020).

Contradictory to other studies in Africa which reported reduced abundance in the *Actinobacteriota* phylum (Zoetendal et al., 2008; Filippo et al., 2017; Costea et al., 2018), interestingly, in this study, we observed an abundance in the *Actinobacteriota* phylum (9.5%) in the healthy individuals, making it the second most abundant phylum in healthy individuals in this study (**Supplementary Figure 1**). The abundance of *Actinobacteriota* was driven mainly by the *Bifidobacterium* genera (**Figure 1A** and **Supplementary Table 2**) which has been shown to be among the first microbes to colonize the human gut and very beneficial in gut homeostasis and digestion of fiber to produce SCFAs (O’Callaghan and Sinderen, 2016). Contrarily, in all the TB cohorts, *Actinobacteria* was significantly decreased compared to the controls (**Supplementary Figure 1**).

Similar to other studies which associated *Prevotella* with high fiber-carbohydrate diet mostly in rural areas in sub-Saharan (Filippo et al., 2010; Parbie et al., 2021a), we observed an abundance in the genera *Prevotella* as the dominant group of phylum *Bacteriodota* in healthy individuals. Among the TB cohorts, *Prevotella* was more abundant in the TB-DM cohorts (3.4%) (**Supplementary Table 2**) allowing for some association with studies that recorded enrichment of this genera in metabolic disorders such as type 2 diabetes (Precup and Vodnar, 2019; Ding et al., 2022). Also, in addition to *Prevotella*, in the TB-DM cohort, the *Bacteriodes* which has been reported to be positively associated with diabetes (Murphy et al., 2017; Wu et al., 2017), was significantly differentially abundant in the TB-DM cohort (**Figure 6**).

Irrespective of the overall decrease in microorganism of the *Firmicutes*, *Bacteroidetes* and *Actinobacteria* phyla in all TB cohorts compared to healthy individuals, an enrichment of diverse commensal bacteria of the phylum *Proteobacteria* containing potential pathogenic bacteria and inflammatory related organisms and other pathobionts was observed. Specifically, the genera *Escherichia*- *Shigella*, documented to be abundant in a Ghanaian cohort (Parbie et al., 2021a), as well as other African populations (de Filippo et al., 2017; Mancabelli et al., 2017). Interestingly, in this study, *Escherichia*- *Shigella* was not detected as a predominant bacterium in the gut of healthy individuals (**Figures 1A, B** and **Supplementary Figure 1**). This may be attributable to difference in population dynamics including socioeconomic status and dietary habits; the participants in the present study were from urban communities in Accra, the capital city of Ghana, compared to participants from “peri-urban” communities in the previous study (Parbie et al., 2021a). Also, a study in Korea reported an abundance of the *Proteobacteria* phylum in type 2 diabetes individuals (Shin et al., 2015) but no such association was found in this study. We however observed a high abundance of *Proteobacteria* in TB-HIV individuals (**Supplementary Table 2**) driven mostly by the *Escherichia*- *Shigella* family. This genera have been associated with high inflammatory activities as seen in tuberculosis allowing for proliferation of inflammatory-related microorganisms (Eribo et al., 2020) which may not cause disease itself but rather serve to increase susceptibility to intestinal inflammation (Zeng et al., 2017). Additionally, abundances of *Protobacteria* have been associated with translocation and inflammation of HIV infected individuals (Parbie et al., 2021b).

Intensive phase anti-TB therapy comprises drugs with specific TB targets (HZE) and a broad-spectrum antibiotics (R) (Jnawali and Ryoo, 2013). Broad spectrum antibiotics such as RIF, which are active against most bacterial pathogens, could be responsible for the antibiotic associated perturbations (Dethlefsen and Relman, 2011).

After HRZE treatment, there was a higher abundance of bacteria groups in all the follow-up groups, especially in the TB-HIV cohort (**Figure 4B**). There was an overall enrichment in the genera *Veillonella*, *Erysipelatoclostridium*, and *Fusobacterium* at follow-up compared to the baseline (**Figure 8**), like findings from a study reporting abundance of *Erysipelatoclostridium* in a United States population after HRZE treatment (Wipperman et al., 2017). The genera *Erysipelatoclostridium* and *Fusobacterium* are known to be abundant during inflammatory and immune reducing conditions as in TB patients as well as in HIV infection and diabetes (Neuhaus et al., 2010; Etna et al., 2014). While *Erysipelatoclostridium* possess the ability to flourish during broad-spectrum antibiotic treatment (Dinh et al., 2014), *Fusobacterium* produces chemical signals that can induce apoptosis in monocytes and macrophages through activation of free fatty acid receptors (Lee et al., 2018; Gu et al., 2020), which is also observed in gut mucosa of colorectal cancer patients (Kelly et al., 2018). Even though the significance of HRZE induced taxonomic perturbations is not well-understood, the majority of these bacteria have been associated with immune-inducing phenotypes relevant for TB immunity (Wipperman et al., 2017).

Contrary, to previous reports which identified vitamin B12 deficiency as a serious problem in diabetics (Liu et al., 2006; Pflipsen et al., 2009; Kibirige and Mwebaze, 2013) due to a negative relationship with metformin, this was not observed in our study population. We found a significant abundance of methylmalonyl-CoA mutase in the gut of TB-DM patients compared to controls. This enzyme is involved in Vitamin B12 synthesis, and degradation of amino acids, short-chain fatty acids and cholesterol (Blum et al., 2021). Thus, the abundance of this enzyme and MTB’s capacity for *de novo* biosynthesis of vitamin B12 (Gopinath et al., 2013), resulting in reports of elevated B12 vitamin in TB patients (Gebremicael et al., 2019), could account for our observation. The abundance of methylmalonyl-CoA mutase in only TB-DM group is intriguing and could be the body’s homeostasis activity (Takahashi et al., 1994). Also, a significant abundance of organisms involved in isopropanol biosynthesis in the TB-DM cohort was intriguing, since the metabolite produced is known to inhibit growth of *Mycobacterium tuberculosis*. However, this abundance was only detected in TB-DM cohort and not in the other TB groups. Additionally, an increase of L-rhamnose mutarotase which is involved in the L-rhamnose metabolism pathway for carbohydrate metabolism (Ryu et al., 2005), and very crucial in the diabetes control (Solis-Herrera et al., 2021), was abundant in the TB-DM cohorts.

We acknowledge some limitations especially in the sample size of our control and TB-HIV cohort. However due to the nature of our study, we believe the best representation of healthy controls would be those individuals that live in the same household as the test subjects to rule out any bias. But this number was fewer than expected and we were unable to use previously published gut microbiome data from Ghana as reference gut microbiome due to difference in population dynamics including socioeconomic status, dietary habits, and geographical location. Nevertheless our study reveals a profound impact of TB on the gut microbiome.

## Conclusion

This study describes a profound gut dysbiosis in TB patients with or without diabetes or HIV comorbidities compared to the healthy controls. Several opportunistic pathogens of the bacteria groups including *Escherichia-shigella*, *Streptococcus* and *Enterococcus* were enriched in all TB cohorts, whereas beneficial bacteria groups such as SCFA-producing bacteria were depleted. Notably, there was significant alteration in microbial population in TB-DM individuals driven mainly by *Bacteriodes*, *Blautia* and *Escherichia-shigella.* Further studies focused at understanding the long-term changes in the gut microbiome of comorbid groups during the entire TB treatment, and its potential impact on the treatment outcome will provide further insight on the role of gut microbiome on progression of TB.

## Data availability statement

The data presented in the study are deposited in the NCBI repository, accession number PRJNA876282.

## Ethics statement

The studies involving human participants were reviewed and approved by Institutional Review Board of the Noguchi Memorial Institute for Medical Research with Federal wide Assurance number FWA00001824. The patients/participants provided their written informed consent to participate in this study.

## Author contributions

PM, AB, IL, and EA contributed to sample collection and possessing. PM, AA-P, IO, SA, and DY-M contributed to the methodology. PM, DN, IL, CG, EA, SA contributed to the investigation. PM, PP, and IO did formal analysis. PM, DN, and CG performed all laboratory experiments. PM prepared the original draft of the manuscript. PM, PP, AB, PA, AA-P, IO, SA, and DN contributed to reviewing and editing manuscript. IO, AA-P, and DY-M supervised the work. DY-M provided recourses and conceptualized the study. All authors contributed to the article and approved the submitted version.
