# Supplementary material for: Gut microbiome variation in pulmonary TB patients with diabetes or HIV comorbidities
Source: Front Microbiomes. 2023 Mar 15;2:1123064. doi: 10.3389/frmbi.2023.1123064 (PMC12993506; doi:10.3389/frmbi.2023.1123064)
Supplement: Supplementary file 1 [file DataSheet_1.pdf]

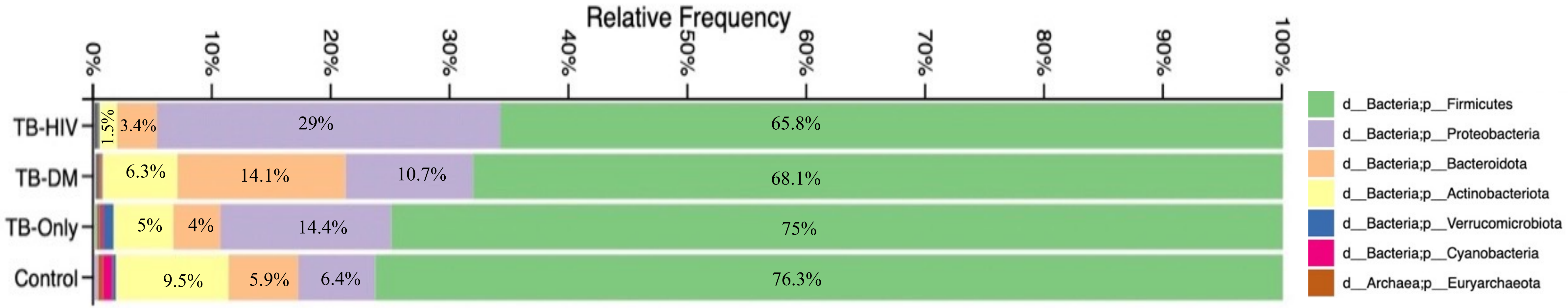

Supplementary Figure 1: Relative frequency of the of the four most abundant phyla in TB-Only, TB-DM, TB-HIV, and healthy controls. *Firmicutes* was the most dominant phyla across all groups, with high frequency in *Proteobacteria* in TB groups and *Bacteroidota* in TB-DM groups.
