# Supplementary material for: Gut microbiome variation in pulmonary TB patients with diabetes or HIV comorbidities
Source: Front Microbiomes. 2023 Mar 15;2:1123064. doi: 10.3389/frmbi.2023.1123064 (PMC12993506; doi:10.3389/frmbi.2023.1123064)
Supplement: Supplementary file 2 [file DataSheet_2.pdf]

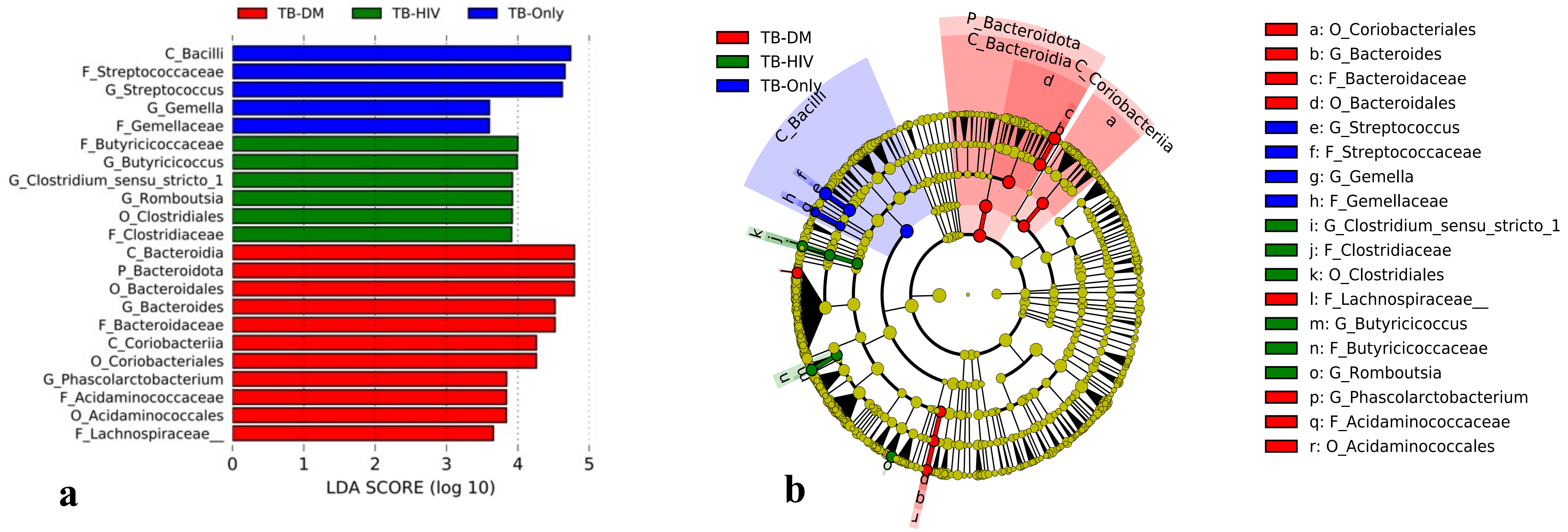

Supplementary Figure 2: Difference in abundant taxa between TB-only, TB-DM and TB-HIV (a) LDA and LEfSe at 3.6 threshold and alpha of 0.01 **(b)** Cladogram of all TB cohorts**.** High abundance of genera *Streptococcus* and *Gamella* in TB-only with *Butyricicoccus* and *Romboutsia* in TB-HIV and *Bacteriodes* , *Phascolarctobacterium* in TB-DM groups.
