# Supplementary material for: Gut microbiome variation in pulmonary TB patients with diabetes or HIV comorbidities
Source: Front Microbiomes. 2023 Mar 15;2:1123064. doi: 10.3389/frmbi.2023.1123064 (PMC12993506; doi:10.3389/frmbi.2023.1123064)
Supplement: Supplementary file 3 [file DataSheet_3.pdf]

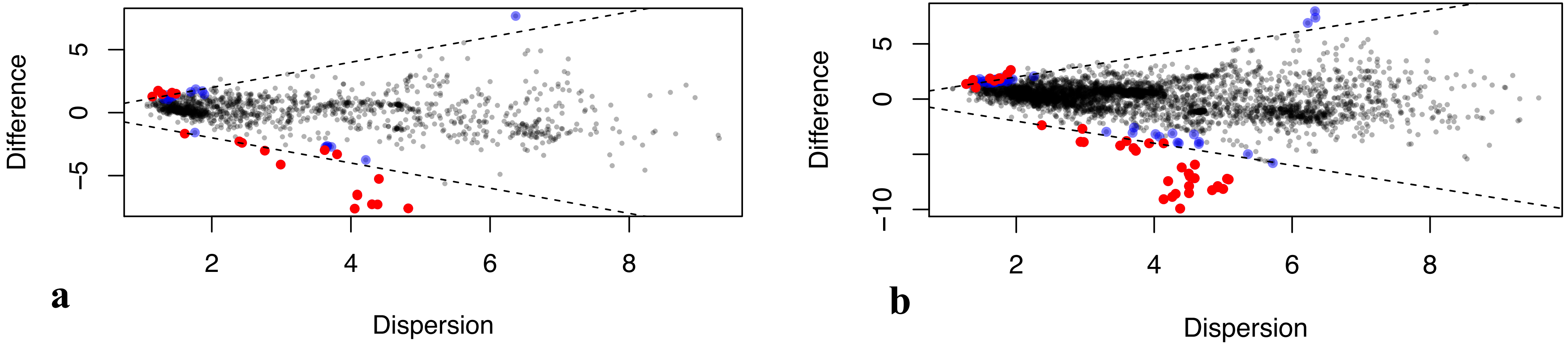

Supplementary Figure 3: Predicting functional abundances based on marker gene sequences. Differential abundance using Welch's t-test and Wilcoxon rank test for features between TB-DM and healthy control group. Significant features by both test in red, features by one test in blue, non-significant rare features in black and abundant in grey **(a)** Fold-change to variance (MW) plots of Enzyme classification (EC) numbers **(b)** KEGG ortholog (KO) MW plot
