# Supplementary material for: Gut microbiome variation in pulmonary TB patients with diabetes or HIV comorbidities
Source: Front Microbiomes. 2023 Mar 15;2:1123064. doi: 10.3389/frmbi.2023.1123064 (PMC12993506; doi:10.3389/frmbi.2023.1123064)
Supplement: Supplementary file 5 [file DataSheet_5.pdf]

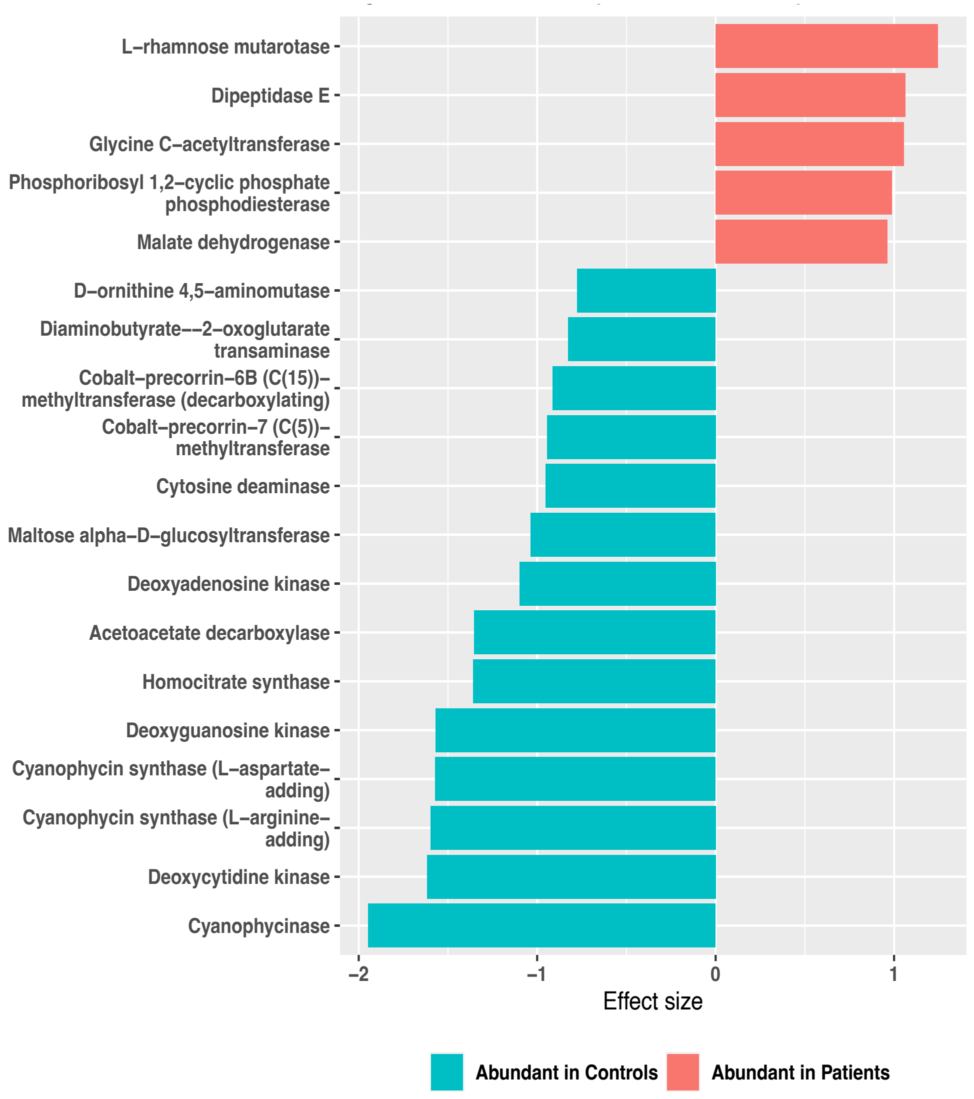

Supplementary Figure 5: Significant differentially abundant predictive Enzyme classification groups. PICRUSt2 was used to determine the predictive microbial metagenome functional abundances from 16S rRNA sequences. Predicted functional abundances were analyzed with ALDEx2 to determine differentially abundant enzyme classification.
