# Supplementary material for: Gut microbiome variation in pulmonary TB patients with diabetes or HIV comorbidities
Source: Front Microbiomes. 2023 Mar 15;2:1123064. doi: 10.3389/frmbi.2023.1123064 (PMC12993506; doi:10.3389/frmbi.2023.1123064)
Supplement: Supplementary file 6 [file Table_1.docx]

**Supplementary Table 1: Sequencing metadata**

| \| **Sequencing code** \| **Category** \| **Age** \| **Sex** \| **Recruitment type** \| **TB Bacterial Load** \| \| --- \| --- \| --- \| --- \| --- \| --- \| \| 1A \| TB-DM \| 41 \| M \| Baseline \| High \| \| 2A \| TB-DM \| 50 \| F \| Follow up \| Neg \| \| 3A \| TB-DM \| 46 \| M \| Baseline \| High \| \| 4A \| TB-DM \| 67 \| M \| Baseline \| Medium \| \| 5A \| TB-DM \| 53 \| M \| Follow up \| Neg \| \| 6A \| TB-DM \| 55 \| M \| Baseline \| High \| \| 7A \| TB-Only \| 41 \| M \| Baseline \| High \| \| 8A \| TB-DM \| 68 \| F \| Baseline \| High \| \| 9A \| TB-DM \| 52 \| M \| Follow up \| Neg \| \| 10A \| TB-DM \| 58 \| M \| Follow up \| Neg \| \| 11A \| TB-Only \| 33 \| F \| Baseline \| Medium \| \| 12A \| TB-DM \| 53 \| M \| Baseline \| High \| \| 13A \| TB-DM \| 60 \| F \| Baseline \| High \| \| 14A \| TB-DM \| 50 \| F \| Baseline \| High \| \| 15A \| TB-DM \| 61 \| M \| Follow up \| Neg \| \| 16A \| TB-DM \| 38 \| F \| Follow up \| Neg \| \| 17A \| TB-DM \| 38 \| F \| Baseline \| High \| \| 18A \| TB-DM \| 52 \| M \| Follow up \| Neg \| \| 19A \| TB-DM \| 58 \| M \| Follow up \| Neg \| \| 20A \| Control \| 54 \| M \| Control \| Neg \| \| 21A \| TB-DM \| 55 \| M \| Baseline \| High \| \| 22A \| TB-DM \| 58 \| M \| Baseline \| Medium \| \| 23A \| TB-DM \| 61 \| M \| Baseline \| High \| \| 24A \| TB-Only \| 18 \| M \| Follow up \| Neg \| \| 25A \| TB-Only \| 14 \| M \| Follow up \| Neg \| \| 26A \| TB-Only \| 37 \| M \| Follow up \| Neg \| \| 27A \| TB-Only \| 26 \| M \| Follow up \| Neg \| \| 28A \| TB-Only \| 20 \| M \| Follow up \| Neg \| \| 29A \| TB-Only \| 22 \| F \| Follow up \| Neg \| \| 30A \| TB-Only \| 49 \| M \| Follow up \| Neg \| \| 31A \| TB-Only \| 20 \| M \| Baseline \| High \| \| 32A \| TB-HIV \| 26 \| F \| Baseline \| High \| \| 33A \| TB-Only \| 50 \| F \| Baseline \| High \| \| 34A \| TB-HIV \| 48 \| M \| Baseline \| Low -Rif resistant \| \| 35A \| TB-DM \| 52 \| M \| Baseline \| High \| \| 36A \| TB-HIV \| 48 \| M \| Follow up \| Neg \| \| 37A \| TB-HIV \| 41 \| M \| Baseline \| Low \| \| 38A \| TB-Only \| 41 \| M \| Baseline \| High \| \| 39A \| TB-HIV \| 37 \| F \| Baseline \| High-rif resistance \| \| 40A \| TB-Only \| 44 \| M \| Baseline \| Low \| \| 41A \| TB-Only \| 30 \| M \| Baseline \| High \| \| 42A \| TB-Only \| 64 \| M \| Baseline \| High \| \| 43A \| TB-Only \| 33 \| M \| Baseline \| Low \| \| 44A \| TB-Only \| 55 \| M \| Baseline \| High \| \| 45A \| TB-Only \| 30 \| M \| Baseline \| Medium \| \| 46A \| TB-Only \| 37 \| M \| Follow up \| Neg \| \| 47A \| TB-Only \| 51 \| F \| Follow up \| Neg \| \| 48A \| TB-Only \| 51 \| F \| Follow up \| Neg \| \| 50A \| TB-Only \| 39 \| M \| Baseline \| High \| \| 51A \| TB-Only \| 64 \| M \| Baseline \| High \| \| 52A \| TB-Only \| 64 \| M \| Follow up \| Neg \| \| 53A \| TB-Only \| 28 \| M \| Follow up \| Neg \| \| 54A \| TB-Only \| 33 \| M \| Follow up \| Neg \| \| 55A \| TB-Only \| 26 \| M \| Baseline \| Low \| \| 56A \| TB-Only \| 20 \| F \| Follow up \| Neg \| \| 57A \| Control \| 39 \| F \| Control \| Neg \| \| 60A \| TB-Only \| 61 \| M \| Follow up \| Neg \| \| 62A \| TB-Only \| 22 \| M \| Follow up \| Neg \| \| 63A \| TB-Only \| 66 \| F \| Follow up \| Neg \| \| 64A \| Control \| 19 \| M \| Control \| Neg \| \| 65A \| TB-Only \| 40 \| M \| Follow up \| Neg \| \| 66A \| TB-DM \| 53 \| M \| Baseline \| High \| \| 67A \| TB-Only \| 58 \| M \| Baseline \| Low \| \| 68A \| TB-Only \| 28 \| M \| Baseline \| Medium \| \| 69A \| TB-Only \| 28 \| M \| Baseline \| High \| \| 70A \| TB-Only \| 44 \| M \| Follow up \| Neg \| \| 71A \| TB-Only \| 44 \| M \| Baseline \| Low \| \| 72A \| TB-Only \| 61 \| M \| Follow up \| Neg \| \| 73A \| TB-Only \| 61 \| M \| Baseline \| High \| \| 74A \| TB-Only \| 67 \| M \| Follow up \| Neg \| \| 75A \| TB-Only \| 22 \| F \| Baseline \| High \| \| 76A \| TB-DM \| 55 \| M \| Baseline \| High \| \| 77A \| TB-Only \| 33 \| M \| Baseline \| Medium \| \| 78A \| TB-Only \| 48 \| F \| Baseline \| Medium \| \| 79A \| TB-Only \| 50 \| M \| Baseline \| Medium \| \| 80A \| TB-Only \| 50 \| M \| Follow up \| Neg \| \| 81A \| Control \| 28 \| M \| Control \| Neg \| \| 82A \| TB-Only \| 52 \| M \| Baseline \| High \| \| 83A \| TB-Only \| 39 \| M \| Follow up \| Neg \| \| 84A \| Control \| 29 \| F \| Control \| Neg \| \| 85A \| TB-Only \| 19 \| F \| Baseline \| Medium \| \| 86A \| TB-Only \| 56 \| F \| Baseline \| High \| \| 87A \| Control \| 36 \| F \| Control \| Neg \| \| 88A \| TB-Only \| 21 \| M \| Baseline \| High \| \| 89A \| TB-Only \| 21 \| M \| Baseline \| Low \| \| 90A \| TB-Only \| 31 \| M \| Follow up \| High \| \| 91A \| TB-Only \| 31 \| M \| Baseline \| High \| \| 92A \| TB-Only \| 54 \| M \| Baseline \| High \| \| 93A \| TB-Only \| 31 \| M \| Baseline \| High \| \| 94A \| TB-Only \| 33 \| F \| Follow up \| Neg \| \| 95A \| TB-DM \| 58 \| F \| Baseline \| Low \| \| 96A \| TB-Only \| 26 \| M \| Baseline \| Medium \| \| 97A \| TB-Only \| 49 \| M \| Baseline \| High \| \| 98A \| TB-DM \| 45 \| M \| Baseline \| High \| \| 99A \| TB-DM \| 40 \| M \| Baseline \| Medium \| \| 1B \| TB-DM \| 52 \| M \| Baseline \| Low \| \| 2B \| TB-Only \| 26 \| F \| Baseline \| High \| \| 3B \| TB-Only \| 25 \| M \| Follow up \| Low -Rif resistant \| \| 4B \| TB-Only \| 29 \| F \| Baseline \| High \| \| 5B \| Control \| 35 \| F \| Control \| Neg \| \| 6B \| TB-Only \| 37 \| M \| Baseline \| Low \| \| 7B \| TB-Only \| 67 \| M \| Baseline \| Medium \| \| 8B \| TB-Only \| 18 \| M \| Baseline \| High \| \| 9B \| TB-Only \| 20 \| M \| Baseline \| High \| \| 10B \| TB-Only \| 20 \| F \| Baseline \| Medium \| \| 11B \| TB-DM \| 33 \| M \| Baseline \| Low \| \| 12B \| TB-DM \| 50 \| M \| Baseline \| High \| \| 13B \| TB-Only \| 25 \| M \| Baseline \| High \| \| 14B \| TB-HIV \| 39 \| M \| Baseline \| Low \| \| 15B \| TB-Only \| 32 \| M \| Baseline \| High \| \| 16B \| TB-Only \| 59 \| M \| Baseline \| High \| \| 17B \| TB-DM \| 67 \| M \| Follow up \| Neg \| \| 18B \| TB-Only \| 43 \| F \| Baseline \| High \| \| 19B \| TB-HIV \| 60 \| M \| Baseline \| Low \| \| 20B \| TB-Only \| 66 \| M \| Follow up \| Neg \| \| 21B \| Control \| 53 \| F \| Control \| Neg \| \| 22B \| TB-Only \| 47 \| F \| Follow up \| Neg \| \| 23B \| TB-Only \| 21 \| M \| Follow up \| Neg \| \| 24B \| TB-Only \| 45 \| M \| Baseline \| High \| \| 25B \| TB-Only \| 46 \| M \| Follow up \| Neg \| \| 26B \| Control \| 31 \| F \| Control \| Neg \| \| 27B \| TB-Only \| 28 \| M \| Follow up \| Neg \| \| 28B \| TB-Only \| 19 \| F \| Baseline \| High \| \| 29B \| Control \| 20 \| F \| Control \| Neg \| \| 30B \| TB-DM \| 40 \| M \| Follow up \| Neg \| \| 31B \| TB-HIV \| 23 \| F \| Follow up \| Neg \| \| 32B \| TB-HIV \| 41 \| M \| Follow up \| Neg \| \| 33B \| TB-HIV \| 30 \| F \| Follow up \| Neg \| \| 34B \| TB-Only \| 28 \| M \| Baseline \| High \| \| 35B \| TB-Only \| 70 \| M \| Baseline \| Neg \| \| 36B \| TB-Only \| 47 \| F \| Baseline \| High \| \| 37B \| TB-Only \| 66 \| M \| Baseline \| High \| \| 38B \| TB-HIV \| 56 \| M \| Follow up \| Neg \| \| 39B \| TB-Only \| 18 \| M \| Baseline \| Medium \| \| 41B \| TB-Only \| 37 \| M \| Baseline \| Neg \| \| 42B \| TB-Only \| 45 \| M \| Follow up \| Neg \| \| 43B \| TB-DM \| 58 \| M \| Baseline \| Medium \| \| 44B \| TB-Only \| 56 \| M \| Baseline \| High \| \| 45B \| TB-Only \| 40 \| M \| Baseline \| Low \| \| 46B \| TB-HIV \| 29 \| M \| Baseline \| Medium \| \| 48B \| TB-Only \| 64 \| M \| Baseline \| High \| \| 49B \| TB-DM \| 55 \| M \| Baseline \| High \| \| 51B \| TB-Only \| 26 \| F \| Baseline \| High \| \|  \|  \|  \|  \|  \|  \| |
| --- | --- | --- | --- | --- | --- | --- | --- | --- | --- | --- | --- | --- | --- | --- | --- | --- | --- | --- | --- | --- | --- | --- | --- | --- | --- | --- | --- | --- | --- | --- | --- | --- | --- | --- | --- | --- | --- | --- | --- | --- | --- | --- | --- | --- | --- | --- | --- | --- | --- | --- | --- | --- | --- | --- | --- | --- | --- | --- | --- | --- | --- | --- | --- | --- | --- | --- | --- | --- | --- | --- | --- | --- | --- | --- | --- | --- | --- | --- | --- | --- | --- | --- | --- | --- | --- | --- | --- | --- | --- | --- | --- | --- | --- | --- | --- | --- | --- | --- | --- | --- | --- | --- | --- | --- | --- | --- | --- | --- | --- | --- | --- | --- | --- | --- | --- | --- | --- | --- | --- | --- | --- | --- | --- | --- | --- | --- | --- | --- | --- | --- | --- | --- | --- | --- | --- | --- | --- | --- | --- | --- | --- | --- | --- | --- | --- | --- | --- | --- | --- | --- | --- | --- | --- | --- | --- | --- | --- | --- | --- | --- | --- | --- | --- | --- | --- | --- | --- | --- | --- | --- | --- | --- | --- | --- | --- | --- | --- | --- | --- | --- | --- | --- | --- | --- | --- | --- | --- | --- | --- | --- | --- | --- | --- | --- | --- | --- | --- | --- | --- | --- | --- | --- | --- | --- | --- | --- | --- | --- | --- | --- | --- | --- | --- | --- | --- | --- | --- | --- | --- | --- | --- | --- | --- | --- | --- | --- | --- | --- | --- | --- | --- | --- | --- | --- | --- | --- | --- | --- | --- | --- | --- | --- | --- | --- | --- | --- | --- | --- | --- | --- | --- | --- | --- | --- | --- | --- | --- | --- | --- | --- | --- | --- | --- | --- | --- | --- | --- | --- | --- | --- | --- | --- | --- | --- | --- | --- | --- | --- | --- | --- | --- | --- | --- | --- | --- | --- | --- | --- | --- | --- | --- | --- | --- | --- | --- | --- | --- | --- | --- | --- | --- | --- | --- | --- | --- | --- | --- | --- | --- | --- | --- | --- | --- | --- | --- | --- | --- | --- | --- | --- | --- | --- | --- | --- | --- | --- | --- | --- | --- | --- | --- | --- | --- | --- | --- | --- | --- | --- | --- | --- | --- | --- | --- | --- | --- | --- | --- | --- | --- | --- | --- | --- | --- | --- | --- | --- | --- | --- | --- | --- | --- | --- | --- | --- | --- | --- | --- | --- | --- | --- | --- | --- | --- | --- | --- | --- | --- | --- | --- | --- | --- | --- | --- | --- | --- | --- | --- | --- | --- | --- | --- | --- | --- | --- | --- | --- | --- | --- | --- | --- | --- | --- | --- | --- | --- | --- | --- | --- | --- | --- | --- | --- | --- | --- | --- | --- | --- | --- | --- | --- | --- | --- | --- | --- | --- | --- | --- | --- | --- | --- | --- | --- | --- | --- | --- | --- | --- | --- | --- | --- | --- | --- | --- | --- | --- | --- | --- | --- | --- | --- | --- | --- | --- | --- | --- | --- | --- | --- | --- | --- | --- | --- | --- | --- | --- | --- | --- | --- | --- | --- | --- | --- | --- | --- | --- | --- | --- | --- | --- | --- | --- | --- | --- | --- | --- | --- | --- | --- | --- | --- | --- | --- | --- | --- | --- | --- | --- | --- | --- | --- | --- | --- | --- | --- | --- | --- | --- | --- | --- | --- | --- | --- | --- | --- | --- | --- | --- | --- | --- | --- | --- | --- | --- | --- | --- | --- | --- | --- | --- | --- | --- | --- | --- | --- | --- | --- | --- | --- | --- | --- | --- | --- | --- | --- | --- | --- | --- | --- | --- | --- | --- | --- | --- | --- | --- | --- | --- | --- | --- | --- | --- | --- | --- | --- | --- | --- | --- | --- | --- | --- | --- | --- | --- | --- | --- | --- | --- | --- | --- | --- | --- | --- | --- | --- | --- | --- | --- | --- | --- | --- | --- | --- | --- | --- | --- | --- | --- | --- | --- | --- | --- | --- | --- | --- | --- | --- | --- | --- | --- | --- | --- | --- | --- | --- | --- | --- | --- | --- | --- | --- | --- | --- | --- | --- | --- | --- | --- | --- | --- | --- | --- | --- | --- | --- | --- | --- | --- | --- | --- | --- | --- | --- | --- | --- | --- | --- | --- | --- | --- | --- | --- | --- | --- | --- | --- | --- | --- | --- | --- | --- | --- | --- | --- | --- | --- | --- | --- | --- | --- | --- | --- | --- | --- | --- | --- | --- | --- | --- | --- | --- | --- | --- | --- | --- | --- | --- | --- | --- | --- | --- | --- | --- | --- | --- | --- | --- | --- | --- | --- | --- | --- | --- | --- | --- | --- | --- | --- | --- | --- | --- | --- | --- | --- | --- | --- | --- | --- | --- | --- | --- | --- | --- | --- | --- | --- | --- | --- | --- | --- | --- | --- | --- | --- | --- | --- | --- | --- | --- | --- | --- | --- | --- | --- | --- | --- | --- | --- | --- | --- | --- | --- | --- | --- | --- | --- | --- | --- | --- | --- | --- | --- | --- | --- | --- | --- | --- | --- | --- | --- | --- | --- | --- | --- | --- | --- | --- | --- | --- | --- | --- | --- | --- | --- | --- | --- | --- | --- | --- | --- | --- | --- | --- | --- | --- | --- | --- | --- | --- | --- | --- | --- | --- | --- | --- | --- | --- | --- | --- | --- | --- | --- | --- | --- | --- | --- | --- | --- | --- | --- | --- | --- | --- | --- | --- | --- | --- | --- | --- | --- | --- | --- | --- | --- | --- | --- | --- | --- | --- | --- | --- | --- | --- | --- | --- | --- | --- | --- | --- | --- | --- | --- | --- | --- | --- | --- | --- | --- | --- | --- | --- | --- | --- | --- | --- | --- | --- | --- | --- | --- | --- |
