# Supplementary material for: Gut microbiome variation in pulmonary TB patients with diabetes or HIV comorbidities
Source: Front Microbiomes. 2023 Mar 15;2:1123064. doi: 10.3389/frmbi.2023.1123064 (PMC12993506; doi:10.3389/frmbi.2023.1123064)
Supplement: Supplementary file 7 [file Table_2.docx]

| Phylum | Class | Order | Family | Genus | Mean relative abundance (%) | | | |
| --- | --- | --- | --- | --- | --- | --- | --- | --- |
|  |  |  |  |  | **TB-Only** | **TB-TDM** | **TB-HIV** | **Control** |
| *Firmicutes* | *Bacilli* | *Lactobacillales* | *Streptococcaceae* | *Streptococcus* | 9.5 | 2 | 6.1 | 1.6 |
|  |  |  | *Enterococcaceae* | *Enterococcus* | 5.1 | 5.7 | 12.3 | 0.3 |
|  |  | *Erysipelotrichales* | *Erysipelotrichaceae* | *Holdermanella* | 1 | 1.8 | 0.9 | 2.8 |
|  |  |  | *Erysipelatoclostridiaceae* | *Erysipelatoclostridium* | 3.6 | 3.3 | 1 | 0 |
|  | *Clostridia* | *Lachnospirales* | *Lachnospiraceae* | *Blautia* | 6.5 | 8.6 | 3.3 | 4.8 |
|  |  |  |  | *gnavus_group* | 4.3 | 1.4 | 1.6 | 0 |
|  |  |  |  | *Agathobacter* | 0.7 | 0.6 | 1.6 | 4 |
|  |  |  |  | *Dorea* | 2 | 1 | 1 | 2.7 |
|  | *Clostridia* | *Oscillospirales* | *Ruminococcaceae* | *Faecalibacterium* | 4.6 | 5.8 | 4.9 | 5.7 |
|  |  |  |  | *Subdoligranulum* | 4.7 | 3.5 | 0.8 | 3.6 |
|  |  |  |  | *CAG.352* | 2.9 | 1.3 | 0.9 | 3.9 |
| *Proteobacteria* | *Gammaproteobacteria* | *Enterobacteriales* | *Enterobacteriaceae* | *Escherichia-Shigella* | 11 | 9.2 | 8.7 | 0.9 |
|  |  |  |  | *Klebsiella* | 2.4 | 0.4 | 19.7 | 2.16 |
| *Bacteriodetes* | *Bacteriodia* | *Bacteriodales* | *Prevotellaceae* | *Prevotella* | 1.2 | 3.4 | 2.4 | 2.7 |
|  |  |  | *Bacteriodaceae* | *Bacteriodes* | 1.8 | 7.5 | 2.4 | 0.9 |
| *Actinobacteriota* | *Actinobacteria* | *Bifidobacteriales* | *Bifidobacteriaceae* | *Bifidobacterium* | 1.3 | 1.8 | 0.8 | 4.4 |
|  | Coriobacteriia | Coriobacteriales | Coriobacteriaceae | Collinsella | 0.7 | 0.6 | 1.6 | 2.5 |

**Supplementary Table 2:** Top abundant genera in gut microbiome of the three TB cohorts (TB-only, TB-DM, TB-HIV) and healthy controls
